# Parent-child agreement in different domains of child behavior and health

**DOI:** 10.1371/journal.pone.0231462

**Published:** 2020-04-09

**Authors:** Tanja Poulain, Mandy Vogel, Christof Meigen, Ulrike Spielau, Andreas Hiemisch, Wieland Kiess

**Affiliations:** 1 LIFE Leipzig Research Center for Civilization Diseases, Leipzig University, Leipzig, Sachsen, Germany; 2 Department of Women and Child Health, Hospital for Children and Adolescents and Center for Pediatric Research (CPL), Leipzig University, Leipzig, Sachsen, Germany; 3 Integrated Research and Treatment Center (IFB) Adiposity Diseases, Leipzig University, Leipzig, Sachsen, Germany; Center for Healthy Start Initiative, NIGERIA

## Abstract

**Aim:**

The present study aimed to investigate and compare parent-child agreement in different domains of child health and behavior.

**Methods:**

Data were collected between 2011 and 2019 within the framework of the LIFE Child study (Germany). Different subgroups of 10- to 12-year-old children and their parents (n (max) = 692) completed questionnaires on several health behaviors (diet, media use, physical activity, sleep), parameters of health (behavioral strengths and difficulties, psychosomatic complaints), and school grades. Agreement between child and parent reports was evaluated using weighted kappa coefficients. Furthermore, the frequencies of different types of (dis)agreement (parent report > child report, same response, child report > parent report) were assessed and checked for associations with child or parent gender.

**Results:**

Agreement between child and parent reports varied from low to almost perfect, with the greatest levels of agreement for school grades and organized physical activity, and the lowest for dizziness, sleep duration, and the consumption of potatoes. Child gender had no significant effect on parent-child agreement. In contrast, the findings suggest that parent gender had some effect on agreement levels, with higher agreement for certain psychosomatic complaints when parent reports were completed by the mother, and higher agreement for white bread consumption if they were completed by the father. For some of the questionnaire items (especially those relating to behavioral difficulties and psychosomatic complaints, but also to the consumption of individual food products and mobile phone use), the type of (dis)agreement differed depending on child or parent gender.

**Conclusions:**

The findings suggest that the perceptions and reporting strategies of children and their parents can diverge considerably, in particular for behavior that is not easily observable or measurable.

## Introduction

In clinical practice and research on child development, health-related behaviors and characteristics are often assessed using questionnaires. Depending on various factors, though mainly child age, questionnaires are either completed by the children themselves (self reports) or by a caregiver, usually one of the parents (parent reports). Questionnaires offer many benefits, for instance they offer a quick, easy way to assess an individual’s attitudes. However, how informants interpret questions and perceive the assessed behavior is affected by multiple factors. Furthermore, responses to questions may be subject to different biases, which may affect different respondents to different degrees. For example, (young) children may lack sufficient capacity for self-reflection or the ability to evaluate their own behavior, resulting in inadequate self reports [1]. Furthermore, children might have problems recalling examples of a specific behavior [2,3] or might not be able to determine average values or understand the meaning of response categories. Socially desirable responding, in contrast, might be more pronounced in parents [4]. Parents might also have difficulties reporting on how their children behave during their absence (e.g., at school). Because of differing perceptions and the proneness to different biases, child and parent reports on the same behavior might differ substantially.

Previous studies on parent-child agreement have tended to investigate agreement in one specific domain of inquiry. Furthermore, the samples are often composed of children who are suffering from a specific disease rather than normally developing children. Regarding evaluations of *health behavior*, several studies have investigated agreement for questions of diet, physical activity, media use, or sleep. With respect to children’s diet, the amount of parent-child agreement has been shown to differ depending on the type of food assessed, with low agreement for the consumption of fruits and vegetables [5–7], but slightly higher agreement for sweetened beverages [7,8], and moderate agreement for the consumption of fish, eggs, and fast food [7]. Regarding aspects of eating culture, e.g., having breakfast or sharing dinners, agreement between child and parent reports has been shown to be low to fair [9]. In a study that compared dietary reports completed by 8- to 11-year-old children and their parents with energy expenditure as measured by the doubly labeled water technique revealed that child reports were more accurate than parent reports [10]. Parents, especially mothers, have been shown to overestimate the energy intake of their children [10]. Regarding physical activity or outdoor play, agreement between child and parent reports has been reported to be fair [9,11]. Interestingly, agreement has been shown to be higher for organized than for non-organized physical activity [12]. This finding suggests that agreement for well-defined and regular activities is much higher than for activities that may take place irregularly and without the knowledge of parents. With respect to media use, parent-child agreement has been shown to be low to fair [9,12]. In two studies, children reported longer screen times than their parents [4,12]. In another study, parents reported a higher frequency of TV use than their children [9]. For sleep problems and sleep quality in children, parent-child agreement has been found to be low [13–16]. In two studies, children reported significantly more sleep problems and lower sleep quality than their parents [13,17]. In another study, the frequency of children reporting sleep problems while parents did not (10%) was comparable to the frequency of parents reporting sleep problems while children did not (7%) [16].

Regarding evaluations of *mental/physical health* of children, several studies have investigated parent-child agreement in reporting behavioral difficulties [18–27]. Overall, in these studies, parent-child agreement was reported to be moderate [18–22] to high [23]. Whereas most of the studies observed higher agreement in reporting with regard to externalizing problems [22,24,25], others found higher agreement for internalizing problems [20] or no difference for externalizing and internalizing problem behavior [21]. Where differences between children and parents were observed, children tended to report more behavioral difficulties than their parents [19–21,27], especially when it came to internalizing problems [26,27]. Factors that have been shown to impact the amount of parent-child agreement in reporting behavioral difficulties are parental engagement, socio-economic status (SES) [19,21], problem behavior/illness on the part of the child [1,18,20], and child age [23]. In contrast, levels of parent-child agreement have not been found to differ between girls and boys [1,19]. For other aspects of mental health, parent-child agreement was shown to be low, e.g., for psychosocial functioning [28], (psycho)somatic symptoms [21,29] and anxiety [30–32]. For quality of life, results have been mixed [33–36], suggesting that agreement might differ depending on contextual factors, e.g., the child’s health status.

In contrast to parent-child agreement regarding health behaviors and aspects of physical/mental health, agreement on more concrete behaviors or facts, e.g., means of transportation to school [9] or school grades [37], has been shown to be almost perfect.

Overall, previous studies indicate that reports by children and parents can vary considerably, with the amount of parent-child agreement differing depending on the behavior assessed. Despite the research that has been done on parent-child agreement, there is still a shortage of studies investigating several different behaviors using a large sample of normally developing children. Furthermore, it is still unclear if the amount of parent-child agreement differs depending on the gender of the child or the gender of the parent. The present study investigated parent-child agreement in several behavioral, health and performance-related domains (diet, media use, physical activity, sleep, behavioral strengths and difficulties, psychosomatic complaints, school grades). It also explored associations between parent-child agreement and child or parent gender. Potentially, the findings can reveal the domains in regard to which the perceptions or reporting strategies of children and parents differ most and, consequently, for what areas of inquiry it might be helpful to assess the views of more than one informant. Additionally, the results might show whether levels of parent-child agreement differ between mothers and fathers and, therefore, whether reports from both parents can be considered of equivalent value, or whether it would be helpful to ask mothers rather than fathers to complete parental reports, or vice versa. This question is especially interesting as, today, fathers are more involved in family life and education than before, but still usually spend less time with their children than mothers.

Based on previous findings and the assumption that parent-child agreement is higher if the assessed behaviors or symptoms are concrete, easily observable, and unambiguous [24], we hypothesized high parent-child agreement for school grades and organized physical activity (i.e., for behaviors that are well defined or documented). For diet, media use, physical activity, and behavioral strengths and difficulties (i.e., for visible, but less specified behaviors), we hypothesized low to moderate agreement. The lowest agreement was expected for indicators or behaviors that are difficult to observe or measure such as sleep and psychosomatic complaints. In line with previous study findings [1,19], parent-child agreement was hypothesized not to differ depending on the gender of the child. Given that mothers often interact more with children than fathers, we expected higher agreement between mothers and children than between fathers and children.

## Materials and methods

### Participants and design

The data used for the present analyses were collected as part of the LIFE Child study, an ongoing longitudinal cohort study set up to monitor healthy child development and the development of lifestyle diseases from the prenatal stage to adulthood [38,39]. Participation in the LIFE Child study is open to all children and adolescents who are not suffering from any chronic, chromosomal, or syndromal disease. They are mainly recruited using advertisements at public health centers, schools, and clinics in or near the city of Leipzig, Germany. The study was designed in accordance with the Declaration of Helsinki. Before the study began, it was reviewed and approved by the Ethical Committee at the Medical Faculty of Leipzig University (IRB00001750, Reg. No. 264/10-EK). Informed written consent is obtained from all participants (children and parents).

The LIFE Child study is conducted at the LIFE Child study center in Leipzig, Germany. Data are collected through clinical examinations, interviews, and questionnaires. For participants up to the age of 12, questionnaires are usually completed by one of the child’s parents (parent report). From the age of 10 and upwards, children complete questionnaires on their own (self report). There is therefore a 2-year age range (10–12 years) for which questionnaires are completed separately by the child and a parent. This offers the opportunity to compare data from parent-report and self-report questionnaires about the same 10- to 12-year-old children.

The LIFE Child study is a longitudinal study in which children and parents are invited to participate once per year. However, in the present project, only one study visit was taken into account for each child, i.e., each parent-child dyad was only analyzed once. In the case of multiple visits, one visit was chosen randomly. Furthermore, it is important to note that different questionnaires were assessed in different acquisition periods (between 2011 to 2019). As such, the number of children included in the analyses varied across assessments (from 221 to 692, see Table 1).

**Table 1:** Assessments included in the analyses on parent-child agreement.

Assessment	Time period	N	Child gender	Parent gender	Child age (sd)	SES*
**Health behavior**						
Diet	2017–2019	221	124 (56%) boys 97 (44%) girls	193 (87%) mothers 28 (13%) fathers	11.05 (0.80)	18 (8%) low
						120 (55%) middle
						72 (32%) high
						11 (5%) NA
Media usage	2013–2017	611	321 (53%) boys 290 (47%) girls	445 (73%) mothers 166 (27%) fathers	11.78 (0.71)	44 (7%) low
						259 (43%) middle
						274 (45%) high
						30 (5%) NA
Physical activity	2013–2017	610	321 (53%) boys 289 (47%) girls	444 (73%) mothers 166 (27%) fathers	11.78 (0.71)	44 (7%) low
						258 (43%) middle
						274 (45%) high
						30 (5%) NA
Sleep	2012–2015	461	234 (51%) boys 227 (49%) girls	396 (86%) mothers 65 (14%) fathers	11.44 (0.88)	6 (1%) low
						157 (34%) middle
						276 (60%) high
						22 (5%) NA
**Mental/physical health**						
Behavioral strengths and difficulties	2011–2019	692	377 (54%) boys 315 (46%) girls	512 (74%) mothers 180 (26%) fathers	11.75 (0.76)	56 (8%) low
						306 (44%) middle
						206 (30%) high
						14 (2%) NA
Psychosomatic complaints	2015–2019	379	197 (52%) boys 182 (48%) girls	291 (77%) mothers 88 (23%) fathers	11.76 (0.72)	34 (9%) low
						187 (49%) middle
						137 (36%) high
						17 (4%) NA
**School performance**						
School grades	2012–2017	458	245 (54%) boys 213 (46%) girls	382 (83%) mothers 76 (17%) fathers	11.60 (0.84)	47 (10%) low
						239 (52%) middle
						156 (34%) high
						14 (3%) NA

Each parent-child dyad was analyzed once. The acquisition period illustrates how long a specific questionnaire was part of the LIFE Child program. Depending on who accompanied the child at the day of the study visit, the parent report was completed by either the mother or the father (not by both).

SES = Socio-economic status

NA = not available (missing).

### Measures

The available data allowed a comparison of parent and self reports in the areas of *health behavior* (diet, media use, physical activity, sleep), *mental/physical health* (behavioral strengths and difficulties, psychosomatic complaints), and *school performance* (school grades). The questionnaires and the resulting variables used for further analysis are described below:

#### Diet

Participants’ diets were assessed using the questionnaire Composition and Culture of Eating (CoCu) [40]. The 14 items assess how many portions of different food types children eat per day (fruits/vegetables, unsweetened milk products, sweetened milk products, sweetened beverages, wholegrain bread, and white bread) or per week (meat, fish, potatoes, fried potatoes, rice/noodles, ready-made meals, cakes, and unhealthy snacks). Six answer categories were provided: “no portion”, “max. 1 portion”, “2–3 portions”, “4–5 portions”, “6–7 portions”, and “> 7 portions”.

#### Media usage

Media usage was assessed using a questionnaire (S1 Table) that was originally designed for the German Health Interview and Examination Survey for Children and Adolescents (KiGGS) and has already been used in several projects [41–43]. In this questionnaire, children or their parents are asked to state how many hours per day the child usually spends watching TV/video, and using a computer/the internet, and a mobile phone. Five answer categories were provided: “never”, “30 minutes”, “1–2 hours”, “3–4 hours”, and “more than 4 hours”.

#### Physical activity

The questionnaire on children’s physical activity (S1 Table) comprised questions adapted from the KiGGS study and has already been used in several projects [41,42,44]. It assesses the frequency per week of organized physical activity (i.e., in sports clubs), and non-organized physical activity (i.e., outside sports clubs). Responses are given on a five-graded scale with the answer categories “never”, “less than once”, “1–2 times”, “3–5 times”, and “nearly every day”.

#### Sleep

Sleep problems were assessed using two different questionnaires. Parents completed the German version of the Children’s Sleep Habits Questionnaire (CSHQ) [45,46]. Children completed the German version of the Sleep Self Report (SSR), which is adapted from the CSHQ [15,47]. The agreement between these two questionnaires had already been investigated in a previous study [15]. Here, we focused on two of the questions that are asked in exactly the same way in both questionnaires, one on the frequency of short sleep duration and one on the frequency of sleep onset problems. Answers to these questions were to be given on a three-graded scale. The answer categories were “usually (5–7 times per week)”, “sometimes (2–4 times per week)”, and “rarely (never or once per week)”).

#### Behavioral strengths and difficulties

Behavioral strengths and difficulties were assessed using the parent and child versions of the Strengths and Difficulties Questionnaire (SDQ) [48]. The agreement between parent and child reports completed using the SDQ has already been investigated in several studies [18–20,26,27]. The questionnaire comprises 25 questions on five different scales: emotional problems (e.g., feeling depressed or anxious), conduct problems (e.g., aggressive behavior, tantrums), hyperactivity/inattention (e.g., being unfocused or restless), peer-relationship problems (e.g., playing alone, having no friends), and pro-social behavior (e.g., being helpful, sharing things). For each question, three answer categories were provided: “not true”, “somewhat true”, and “certainly true”. The answers to questions belonging to the same scale were added to produce a sum score between 0 and 10 inclusive. These scores were used as variables of interest for further analysis.

#### Psychosomatic complaints

Psychosomatic complaints were assessed using a questionnaire the researchers adapted from the Giessen Subjective Complaints List for children and adolescents [49]. The eight items assess common psychosomatic complaints in children and adolescents, namely headaches, stomach ache, back pain, depressive mood, irritability, nervousness, problems falling asleep, and dizziness. For each complaint, five possible answer categories were offered: “never/rarely”, “once per month”, “nearly every week”, “several times per week”, and “nearly every day”.

#### School grades

School grades were assessed by asking parents and children to report school grades in Mathematics, German (native language), Physical Education, and the first foreign language documented on the most recent school record. In the German school system, school grades range from 1 to 6, with a lower number indicating better performance (1 = “very good”, 2 = “good”, 3 = “satisfactory”, 4 = “sufficient”, 5 = “deficient”, 6 = “insufficient”).

### Statistical analysis

All analyses were performed using R [50]. The level of agreement between child and parent reports was estimated using weighted Cohen’s kappa coefficients. Kappa rates the observed degree of agreement as corrected for expected agreement under the null hypothesis, and therefore reflects a chance-corrected measure of agreement [51]. The weighted kappa coefficient accounts for different degrees of disagreement on ordinal scales (e.g., a difference between categories 1 and 2 is weighted differently than a difference between categories 1 and 5). The degree of agreement was classified using the ranges and labels set out by Landis and Koch [52]: < 0 = poor, .01 - .20 = slight, .21 - .40 = fair, .41 - .60 = moderate, .61 - .80 = substantial, .81–1.00 = almost perfect. Kappa coefficients were calculated separately for girls versus boys, mothers versus fathers, and for the whole sample.

To give a better overview of the type of (dis)agreement between self (i.e. child) reports and parent reports, we calculated how many children reported the same (e.g., same media usage times), greater (e.g., longer media usage times), or lower (e.g., shorter media usage times) values for the assessed behaviors than their parents. The corresponding (dis)agreement categories were labeled “child = parent”, “child > parent”, and “parent > child”. Using multiple logistic regression, we explored whether the frequency of child reports indicating higher values than the parent reports (i.e., the proportion of parent-child dyads in the “child > parent” category) differed depending on child or parent gender (independent variables).

## Results

### Description of the study samples

Table 1 displays the number of children, mothers, and fathers involved in the different analyses. The distributions of child gender were comparable between the different subsamples and showed a small over-representation of boys (53% versus 47%). As children were more often accompanied by their mothers than by their fathers, parent reports were more frequently completed by mothers than by fathers. In the subsamples on diet, sleep, and school grades, the proportion of fathers was lower (13–17%) than in the other subsamples (23–27%). Regarding child age, the children in the diet subsample were slightly younger overall (M = 11.05) than in the other subsamples (M ranging between 11.44 and 11.75). With respect to the socio-economic status (SES) of participating families, low SES was under-represented and high SES was overrepresented in all subsamples, with an especially unequal distribution in the subsample on sleep (for a more detailed description of how SES is measured in the LIFE Child study see [53,54]). In a representative sample, the distribution of low—middle—high SES would be expected to be 20% - 60% - 20% [54].

### Agreement between self and parent reports

The proportions of children and parents choosing each of the response categories for the various questionnaire items are displayed in S2 Table. Table 2 shows the response categories chosen most frequently by children and parents and the Cohen’s kappa coefficients for the different questionnaire items. For 24% of the items, agreement between children and parents was only slight (some items relating to specific foods and the majority of those relating to psychosomatic complaints). For most of the items (53%), agreement was fair (most of the items for specific foods and for behavioral strengths and difficulties). Moderate agreement was found in 10% of items (e.g. media use). Substantial agreement was only observed for organized PA (3% of total items), and almost perfect agreement was reached for all school grade items (10% of all items).

**Table 2:** Response categories chosen most frequently by children and parents for the various questionnaire items and Cohen’s kappa coefficients (+ 95% CI).

Subject of questionnaire item	Response category chosen most frequently by		Cohen’s Kappa*
	Child	Parent	Coefficient (95% CI)
**Health behavior**			
Diet			
Fruits/vegetables	2-3/day (50%)	2-3/day (54%)	.35 (.21-.51)
Milk unsweetened	1/day (46%)	1/day (59%)	.22 (.09-.34)
Milk sweetened	1/day (63%)	1/day (68%)	.20 (.08-.31)
Sweetened beverages	1/day (40%)	1/day (41%)	.23 (.10-.38)
Wholegrain bread	1/day (42%)	1/day (50%)	.21 (.12-.31)
White bread	1/day (47%)	1/day (49%)	.21 (.07-.34)
Fish	1/ week (51%)	1/ week (68%)	.33 (.22-.44)
Meat	2-3/ week (34%)	4-5/week (32%)	.32 (.23-.40)
Potatoes	1/week (57%)	2-3/week (61%)	.04 (-.07-.16)
Fried potatoes	1/week (62%)	1/week (66%)	.24 (.09-.36)
Rice/noodles	2-3/week (45%)	2-3/week (64%)	.22 (.10-.36)
Ready-made meals	1/week (49%)	1/week (49%)	.33 (.20-.42)
Cakes	1/week (42%)	2-3/week (45%)	.20 (.06-.30)
Unhealthy snacks	2-3/week (42%)	2-3/week (39%)	.10 (-.02-.20)
Media use			
TV	1–2 h/day (52%)	1–2 h/day (52%)	.44 (.38-.53)
Computer	0.5 h/day (41%)	0.5 h/day (48%)	.45 (.37-.53)
Mobile phone	0.5 h/day (33%)	0.5 h/day (36%)	.60 (.53-.65)
Physical activity			
Organized PA	1-2/week (42%)	1-2/week (48%)	.72 (.67-.77)
Non-organized PA	1-2/week (32%)	1-2/week (36%)	.21 (.14-.28)
Sleep			
Short sleep duration	0-1/week (60%)	0-1/week (74%)	.07 (-.03-.16)
Sleep latency problem	0-1/week (37%)	0-1/week (65%)	.22 (.13-.28)
**Mental/physical health**			
Behavioral strengths and difficulties (sum scores)			
Emotional problems	0 (24%)	0 (32%)	.31 (.22-.39)
Conduct problems	1 (33%)	1 (24%)	.32 (.26-.38)
Hyperactivity/inattention	5 (18%)	3 (17%)	.35 (.29-.41)
Peer-relationship problems	1 (23%)	0 (33%)	.38 (.31-.44)
Prosocial behavior	9 (22%)	10 (22%)	.22 (.15-.28)
Psychosomatic complaints			
Headache	Rarely/never (64%)	Rarely/never (49%)	.40 (.30-.50)
Stomach ache	Rarely/never (71%)	Rarely/never (54%)	.50 (.42-.59)
Back pain	Rarely/never (75%)	Rarely/never (74%)	.32 (.22-.43)
Depressive mood	Rarely/never (73%)	Rarely/never (63%)	.16 (.04-.26)
Nervousness	Rarely/never (71%)	Rarely/never (62%)	.13 (-.01-.27)
Irritability	Rarely/never (49%)	1-2/month (34%)	.15 (.05-.24)
Problems falling asleep	Rarely/never (56%)	Rarely/never (50%)	.26 (.18-.36)
Dizziness	Rarely/never (85%)	Rarely/never (92%)	.08 (-.01-.17)
**School grades**			
Mathematics	satisfactory (34%)	satisfactory (33%)	.91 (.89-.94)
German	good (54%)	good (52%)	.88 (.85-.90)
Foreign language	good (51%)	good (49%)	.81 (.77-.86)
Physical Education	good (46%)	good (47%)	.84 (.80-.88)

*The degree of agreement was classified based on ranges/labels set out by Landis and Koch [52]: < 0 = poor, .01 - .20 = slight, .21 - .40 = fair, .41 - .60 = moderate, .61 - .80 = substantial, .81–1.00 = almost perfect.

### Types of (dis)agreement between children and parents

**Fig 1:**
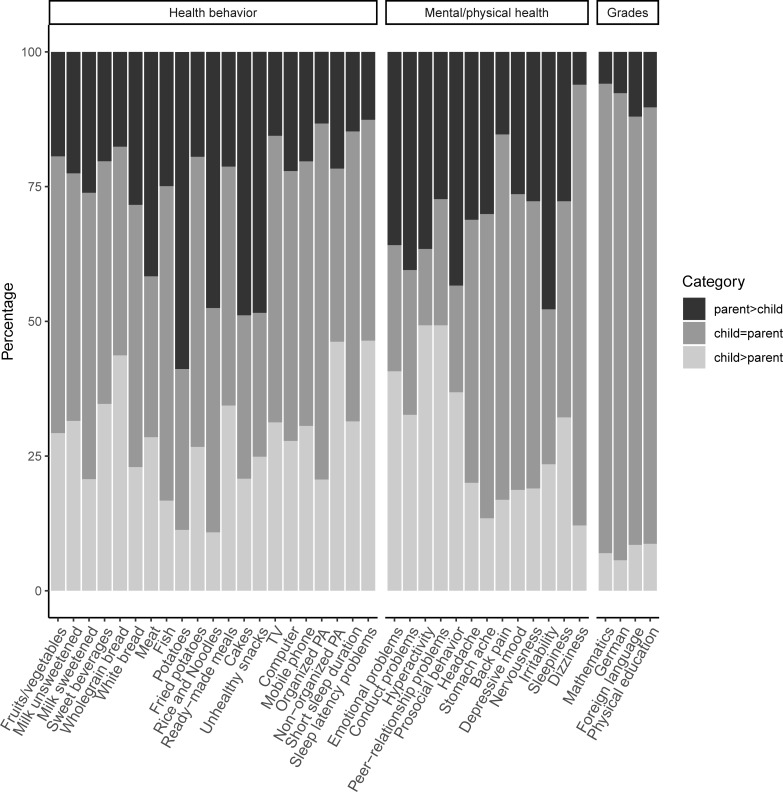
Distribution, for individual questionnaire items, of categories of (dis)agreement between (child) self reports and parent reports.

For a better overview of the categories of agreement/disagreement, for each item we assessed how often children and parents had chosen the same response category (“child = parent”), how often the parent had chosen a higher-scoring response category than the child (“parent > child”), and how often the child had chosen a higher-scoring response category than the parent (“child > parent”). The different distributions of these categories are illustrated in Fig 1.

The frequency of the “child = parent” category varied between 14.16% (for hyperactivity/inattention) and 86.17% (for grades in Mathematics). The frequency of the “parent > child” category was lowest for dizziness (6.07%) and school grades in Mathematics (6.41%) and highest for the consumption of potatoes (58.82%) and cakes (48.87%). The frequency of the “child > parent” category was lowest for grades in German (6.61%) and Mathematics (7.41%) and highest for peer-relationship problems (49.42%) and symptoms of hyperactivity/inattention (49.28%).

Within the two disagreement categories (“child > parent” and “parent > child”), most parent-child dyads (63%) were found to have chosen adjacent response categories (e.g., child selected response category 2 and parent selected response category 1 or 3). The only five items for which the frequency of adjacent responses was below 50% were in the domain of behavioral strengths and difficulties (hyperactivity: 32%, emotional problems: 39%, prosocial behavior: 41%, peer-relationship problems: 45%, conduct problems: 47%).

### Associations between (category of) parent-child agreement and child or parent gender

A comparison of kappa coefficients depending on child gender revealed no significant differences, indicating that parent-child agreement did not differ between girls and boys. However, the analysis of differences in the frequency of the category “child > parent” depending on child gender revealed some significant differences in the domains of health behavior and mental/physical health (see Table 3). For the items relating to emotional problems, symptoms of hyperactivity/inattention, and most psychosomatic symptoms (namely back pain, depressive mood, irritability, problems falling asleep, and dizziness), the category “child > parent” was observed more frequently for dyads including a female child than for those including a male child. For the consumption of fried potatoes and unhealthy snacks, in contrast, this category was observed more frequently with boys than with girls. For all other outcomes, the frequency of the “child > parent” category did not differ significantly between girls and boys (all p > .05).

**Table 3:** Frequency of children choosing a higher response category than their parents broken down by child gender.

	Frequency of category “child > parent”		OR (95% CI)*	p
	Girls	Boys		
**Health behavior**				
Consumption of fried potatoes	18%	34%	0.41 (0.22–0.89)	.007
Consumption of unhealthy snacks	18%	31%	0.48 (0.25–0.92)	.027
**Mental/physical health**				
Emotional problems	47%	35%	1.64 (1.21–2.23)	.002
Hyperactivity/inattention	57%	43%	1.68 (1.24–2.28)	< .001
Back pain	22%	12%	2.04 (1.17–3.55)	.011
Depressive mood	23%	15%	1.77 (1.05–3.00)	.033
Irritability	29%	18%	1.87 (1.15–3.03)	.012
Problems falling asleep	37%	27%	1.60 (1.03–2.47)	.035
Dizziness	18%	7%	2.86 (1.47–5.57)	.002

* Reference = boys. For all other outcomes, the frequency of the category “child > parent” did not differ significantly depending on child gender.

The analysis looking at differences in parent-child agreement depending on parent gender revealed three significant differences. For the consumption of white bread, parent-child-agreement was significantly higher if parent reports were completed by the child’s father (kappa = .73 (95% CI .56 - .89)) rather than the mother (kappa = .13 (95% CI -.02 - .27)). For back pain, in contrast, agreement was significantly higher if the parent report was completed by the child’s mother (kappa = .37 (95% CI .25 - .50)) rather than the father (kappa = .04 (95% CI -.09 - .24)). Similarly, agreement on nervousness was significantly higher if the parent report was completed by the mother (kappa = .20 (95% CI .07 - .37)) rather than the father (kappa = -.12 (95% CI -.25 - .06)).

Table 4 shows the significant differences that emerge in the frequency of the “child > parent” category depending on parent gender. For items relating to certain behavioral difficulties (namely conduct problems, symptoms of hyperactivity/inattention, and peer-relationship problems) and mobile phone use, this category was more frequent among child-mother dyads than among child-father dyads (i.e., the likelihood of the child reporting a higher frequency for these behaviors than the parent was greater if the parent report was completed by the child’s mother). For the consumption of potatoes and for stomach ache, in contrast, this likelihood was higher if the parent report was completed by the father. For all other items, the frequency of the category “child > parent” did not vary significantly depending on parent gender (all p > .05).

**Table 4:** Frequency of children choosing a higher response category than their parents broken down by parent gender.

	Frequency of category “child > parent”		OR (95% CI)*	p
	Mothers	Fathers		
**Health behavior**				
Consumption of potatoes	9%	29%	4.19 (1.60–10.99)	.004
Mobile phone use	33%	24%	0.64 (0.42–0.96)	.031
**Mental/physical health**				
Conduct problems	36%	23%	0.55 (0.37–0.81)	.002
Hyperactivity/inattention	52%	39%	0.59 (0.42–0.84)	.003
Peer-relationship problems	55%	33%	0.40 (0.28–0.57)	< .001
Stomach ache	11%	23%	2.54 (1.36–4.75)	.004

* Reference = mothers. For all other outcomes, the frequency of the category “child > parent” did not differ significantly depending on parent gender.

## Discussion

This study investigated agreement between parent and child reports regarding various behaviors, traits and indicators in the domains of health behavior, mental/physical health, and school performance. Overall, the results demonstrate that the amount of parent-child agreement differs depending on the specific behavior/trait/indicator being assessed and, partly, on parent gender, but not on child gender. As expected, agreement was highest for school grades and organized physical activity, i.e., for well-defined, unambiguous, or well-documented outcomes. For most of the other health behaviors and for behavioral strengths and difficulties, i.e., those that are easily observable but less defined or difficult to measure, parent-child agreement was only fair to moderate. Finally, agreement was lowest for aspects of sleep and most of the assessed psychosomatic complaints, i.e., for behaviors that are difficult to observe or less likely to be observed. The analysis also showed that, where there was disagreement between the child and the parent, in most cases the child and parent chose adjacent response categories.

### Parent-child agreement in the different domains

#### Health behavior

Levels of agreement between child and parent responses regarding the different health behaviors assessed in this study varied between slight and moderate. With regard to diet, the observed agreement was slight to fair, which is in line with previous findings [5–7]. Agreement was particularly low for the consumption of potatoes, with parents (especially mothers) reporting higher consumption levels than the children. For the consumption of white bread, parent-child agreement was higher if parent reports were completed by fathers, which suggests that fathers are more aware of how frequently their children consume this food product. One reason might be that children eat white bread more frequently with their fathers than with their mothers. We also observed that instances of the child reporting higher consumption of unhealthy foods (unhealthy snacks and fried potatoes) than their parent were more common with male children than with female children. A possible reason for this is that girls exhibit a higher underreporting bias than boys [55]. Overall, however, no clear pattern could be identified regarding the type of (dis)agreement. These findings might indicate that both children and parents have difficulties estimating and remembering what they eat. For parents, the estimation might be especially difficult, given that they may not always be aware of what their children eat at school. For children, it might be difficult to judge which foods belong to a specific food group (e.g., which foods are to be counted as potatoes).

Regarding media use, agreement between child and parent reports was moderate and, therefore, higher than for the other health behaviors (with the exception of organized physical activity). It was also higher than the agreement reported in previous studies [9,12]. The greater level of agreement for such behaviors may be due to household rules regulating children’s media use. In line with previous findings, we observed that children tended to report more media use than their parents did [4,12]. Furthermore, the likelihood that the child report indicated more mobile phone use than the parent report was higher if the parent report was completed by the mother (rather than the father). Possible reasons for these findings are that parents (especially mothers) answer in a more socially desirable way [4], and/or do not know how much time their children are spending in front of a screen (e.g., because children have their own media devices and use them without their parents’ knowledge).

With respect to physical activity, parent-child agreement was higher for organized physical activity than non-organized activity. This is in line with the findings from a previous study [12] and the hypothesis that agreement will be higher for well-defined behaviors. For non-organized physical activity, the rather low parent-child agreement observed in the present study is comparable to the agreement reported in previous studies [9,11]. Cases in which children reported more physical activity than their parents did were more frequent than cases in which the parent reported more physical activity. This might indicate that parents do not know to what extent their children are physically active when they are not present. Also, children and parents might have different understandings of how non-organized physical activities are defined, e.g., whether cycling or walking should be considered as physical activity or not.

With regard to sleep, our findings confirm previous studies suggesting low parent-child agreement [13–16]. In line with previous studies [13,17], children tended to report more problems regarding sleep duration and sleep latency than their parents. These discrepancies might be explained by the fact that it is difficult for parents to know when their child has actually fallen asleep, or to judge how much sleep a 10- to 12-year-old child needs. This finding suggests that when investigating sleep difficulties in children and adolescents, it is especially important to consider not only parent reports, but also the reports of the young people themselves.

#### Mental/Physical health

In the domain of mental/physical health, parent-child agreement ranged from slight to moderate. Regarding behavioral strengths and difficulties, the overall agreement between child and parent reports was fair and, therefore, slightly lower than the agreement reported in previous studies [18–23]. In line with other studies [22,24,25], agreement was higher for externalizing problems (and peer-relationship problems) and lower for emotional problems and prosocial behavior. For psychosomatic symptoms, agreement was slight to fair (with the exception of stomach ache for which agreement was moderate), which confirms findings from a previous study [29]. Overall, these findings are in line with the assumption that parent-child agreement is higher for more visible and well-defined behaviors or symptoms (e.g., externalizing behavior) than for behaviors or symptoms that are less obviously visible (e.g., most psychosomatic complaints, emotional problems) [24].

For some of the assessed aspects of mental/physical health, instances of the child reporting more problems/symptoms than their parent were more common with female children than with male children. Several previous studies have shown that girls report more mental health problems than boys [56–58]. The present finding suggests that this gender effect is weaker if indicators of the child’s mental health are estimated by a parent. One might speculate, therefore, that girls tend to estimate their mental state more negatively than boys do, but are less inclined than boys to communicate their problems.

Regarding differences between mothers and fathers, our findings show that parent-child agreement with regard to psychosomatic complaints was higher when the parent report was completed by the child’s mother rather than the father (at least for nervousness and back pain). Furthermore, the likelihood of the child reporting higher levels of psychosomatic problems than their parent was higher if the parent report was completed by the father. One might speculate that psychosomatic complaints are communicated more frequently and more easily to mothers than to fathers, and that this is because they are more often available and considered by the child to be more understanding and less likely to treat the problem as taboo. For behavioral difficulties, in contrast, there was a greater likelihood of the parent reporting more (or at least the same amount of) problems than their children if the parent report was completed by the father, suggesting that fathers may tend to be more ready than mothers to identify certain behaviors as problematic.

#### School grades

Consistent with a previous study [37], parent-child agreement for school grades was almost perfect. The most obvious reason for this high level of agreement is that school grades are objective facts and, as they are usually explicitly documented in school reports, can be remembered easily.

### Strengths and limitations

This study assessed (dis)agreement between child and parent reports in a large German cohort study. The strengths of the study are the sample size and the range and variety of different behaviors and health outcomes investigated. However, some limitations should be acknowledged. One limitation is that the different behaviors/traits/indicators were assessed in different samples of children and over different time periods (with only partial overlap). Furthermore, as we do not have an objective measure of the children’s actual, “true” behavior, the study cannot determine if the child or parent reports are closer to the truth. In regard to parent gender (mothers vs. fathers), it is important to note the overrepresentation of mothers in the sample. Finally, we were not able to investigate reports completed by both parents and a child from the same family. Future studies might include objective measures as reference and compare mothers’ and fathers’ reports for the same child. With a larger sample, the effects of child age might also be explored by including younger and older children and adolescents.

## Conclusions

Child and parent reports can diverge considerably from one another, especially if the behavior or trait is difficult to observe or to estimate objectively. Our results suggest that parent gender may affect the amount of agreement between child and parent reports, but not child gender. Additionally, the type of disagreement (child reports > parent reports or vice versa) is partly affected by child and parent gender, with girls being more likely than boys to report lower values for (mental) health than their parents, and fathers being more likely than mothers to report more behavioral problems and fewer psychosomatic complaints than their children. Overall, the present findings underline the importance of selecting informants carefully and considering potential differences between informants during the process of data interpretation.

## Supporting information

S1 Table Questions of the LIFE Child media use questionnaire and the LIFE Child physical activity questionnaire that were included in the analyses. (DOCX)

S2 Table Frequencies of each response category chosen by children and parents. (DOCX)
