# Supplementary material for: Parent-child agreement in different domains of child behavior and health
Source: PLoS One. 2020 Apr 9;15(4):e0231462. doi: 10.1371/journal.pone.0231462 (PMC7145111; doi:10.1371/journal.pone.0231462)
Supplement: S1 Table — (DOCX) [file pone.0231462.s001.docx]

**S1. Questions of the LIFE Child media use questionnaire and the LIFE Child physical activity questionnaire that were included in the analyses**

Article: Parent-child agreement in different domains of child behavior and health by Tanja Poulain, Mandy Vogel, Christof Meigen, Ulrike Spielau, Andreas Hiemisch, Wieland Kiess (PLOS One)

**Media use**

| **German version (original)** | **English translation** |
| --- | --- |
| Questions | |
| Self report: Wie lange beschäftigst Du dich durchschnittlich am Tag mit folgenden Dingen?   - Fernsehen/Video - Computer/Internet - Handy   Parent report: Wie lange beschäftigt sich Ihr Kind durchschnittlich pro Tag mit folgenden Dingen?   - Fernsehen/Video - Computer/Internet - Handy | Self report: How much time per day do you usually spend using the following items?   - TV/Video - Computer/Internet - Mobile phone   Parent report: How much time per day does your child usually spend using the following items?   - TV/Video - Computer/Internet - Mobile phone |
| Answer categories for TV use, computer use, and mobile phone use: | |
| 1. Gar nicht 2. Ungefähr 30 Minuten pro Tag 3. Ungefähr 1-2 Stunden pro Tag 4. Ungefähr 3-4 Stunden pro Tag 5. Mehr als 4 Stunden pro Tag | 1. Not at all 2. Approximately 30 minutes per day 3. Approximately 1-2 hours per day 4. Approximately 3-4 hours per day 5. More than 4 hours per day |

**Physical activity**

| **German version (original)** | **English translation** |
| --- | --- |
| Questions | |
| Self report: Wie häufig …   - treibst Du Sport in einem Verein? - treibst Du Sport außerhalb eines Vereins?   Parent report: Wie häufig …   - treibt Ihr Kind Sport in einem Verein? - treibt Ihr Kind Sport außerhalb eines Vereins? | Self report: How often …   - do you participate in sports as part of a sports club? - do you participate in sports outside of a sports club?   Parent report: How often …   - does your child participate in sports as part of a sports club? - does your child participate in sports outside of a sports club? |
| Answer categories for organized and non-organized physical activity | |
| 1. Nie 2. Seltener als einmal pro Woche 3. 1-2 mal pro Woche 4. 3-5 mal pro Woche 5. Fast jeden Tag | 1. Never 2. Less than once a week 3. 1-2 times a week 4. 3-5 times a week 5. Almost every day |
