# Supplementary material for: Parent-child agreement in different domains of child behavior and health
Source: PLoS One. 2020 Apr 9;15(4):e0231462. doi: 10.1371/journal.pone.0231462 (PMC7145111; doi:10.1371/journal.pone.0231462)
Supplement: S2 Table — (DOCX) [file pone.0231462.s002.docx]

**S2. Frequencies of each response category chosen by children and parents**

Article: Parent-child agreement in different domains of child behavior and health by Tanja Poulain, Mandy Vogel, Christof Meigen, Ulrike Spielau, Andreas Hiemisch, Wieland Kiess (PLOS One)

**Diet (N = 221)**

|  | Children | | | | | | Parents | | | | | |
| --- | --- | --- | --- | --- | --- | --- | --- | --- | --- | --- | --- | --- |
| Response category | 1 | 2 | 3 | 4 | 5 | 6 | 1 | 2 | 3 | 4 | 5 | 6 |
| Fruits/vegetables^a^ | 4 | 76 | 111 | 26 | 3 | 2 | 4 | 83 | 119 | 12 | 2 | 1 |
| Milk unsweetened^a^ | 31 | 103 | 71 | 15 | 2 | 0 | 24 | 129 | 66 | 1 | 1 | 0 |
| Milk sweetened^a^ | 34 | 139 | 43 | 3 | 3 | 0 | 20 | 150 | 48 | 2 | 1 | 0 |
| Sweetened beverages^a^ | 25 | 89 | 83 | 22 | 2 | 1 | 44 | 92 | 74 | 9 | 2 | 0 |
| Wholegrain bread^a^ | 33 | 93 | 81 | 15 | 0 | 0 | 54 | 112 | 55 | 0 | 0 | 0 |
| White bread^a^ | 33 | 105 | 77 | 7 | 0 | 0 | 24 | 108 | 84 | 4 | 1 | 0 |
| Fish^b^ | 73 | 112 | 32 | 3 | 1 | 0 | 41 | 152 | 26 | 2 | 0 | 0 |
| Meat^b^ | 11 | 45 | 75 | 52 | 28 | 10 | 5 | 30 | 69 | 70 | 35 | 12 |
| Potatoes^b^ | 36 | 127 | 49 | 7 | 1 | 1 | 9 | 55 | 135 | 21 | 1 | 0 |
| Fried potatoes^b^ | 42 | 137 | 36 | 5 | 1 | 0 | 45 | 149 | 26 | 1 | 0 | 0 |
| Rice/noodles^b^ | 1 | 92 | 99 | 25 | 4 | 0 | 27 | 141 | 38 | 13 | 2 | 0 |
| Ready-made meals^b^ | 53 | 108 | 50 | 5 | 4 | 1 | 73 | 108 | 34 | 6 | 0 | 0 |
| Cakes^b^ | 21 | 91 | 80 | 24 | 5 | 0 | 9 | 62 | 101 | 31 | 16 | 2 |
| Unhealthy snacks^b^ | 10 | 59 | 93 | 36 | 17 | 6 | 4 | 42 | 86 | 53 | 24 | 12 |

^a^Response categories: 1 = no portion per day, 2 = max. 1 portion per day, 3 = 2-3 portions per day, 4 = 4-5 portions per day, 5 = 6-7 portions per day, 6 = > 7 portions per day.
^b^Response categories: 1 = no portion per week, 2 = max. 1 portion per week, 3 = 2-3 portions per week, 4 = 4-5 portions per week, 5 = 6-7 portions per week, 6 = > 7 portions per week.

**Media use (N = 611)**

|  | Children | | | | | Parents | | | | |
| --- | --- | --- | --- | --- | --- | --- | --- | --- | --- | --- |
| Response category^a^ | 1 | 2 | 3 | 4 | 5 | 1 | 2 | 3 | 4 | 5 |
| TV/Video | 35 | 166 | 315 | 69 | 26 | 43 | 207 | 320 | 36 | 5 |
| Computer/Internet | 111 | 248 | 185 | 38 | 29 | 92 | 297 | 191 | 29 | 2 |
| Mobile phone | 119 | 201 | 170 | 72 | 49 | 126 | 220 | 182 | 67 | 16 |

^a^Response categories: 1 = never, 2 = 30 minutes per day, 3 = 1-2 hours per day, 4 = 3-4 hours per day, 5 = more than 4 hours per day.

**Physical activity (N = 610)**

|  | Children | | | | | Parents | | | | |
| --- | --- | --- | --- | --- | --- | --- | --- | --- | --- | --- |
| Response category^a^ | 1 | 2 | 3 | 4 | 5 | 1 | 2 | 3 | 4 | 5 |
| Organized | 145 | 40 | 256 | 119 | 50 | 151 | 35 | 291 | 118 | 15 |
| Non-organized | 58 | 161 | 196 | 85 | 110 | 75 | 238 | 219 | 32 | 46 |

^a^Response categories: 1 = never, 2 = 30 minutes per day, 3 = 1-2 hours per day, 4 = 3-4 hours per day, 5 = more than 4 hours per day.

**Sleep (N = 461)**

|  | Children | | | Parents | | |
| --- | --- | --- | --- | --- | --- | --- |
| Response category^a^ | 1 | 2 | 3 | 1 | 2 | 3 |
| Short sleep duration | 45 | 138 | 278 | 15 | 104 | 342 |
| Sleep latency problems | 128 | 163 | 170 | 56 | 107 | 298 |

^a^Response categories: 1 = usually (5-7 times per week), 2 = sometimes (2-4 times per week), 3 = rarely (never or once per week).

**Behavioral strengths and difficulties (N = 692)**

|  | Children | | | | | | | | | | |
| --- | --- | --- | --- | --- | --- | --- | --- | --- | --- | --- | --- |
| Sum score | 0 | 1 | 2 | 3 | 4 | 5 | 6 | 7 | 8 | 9 | 10 |
| Emotional problems | 166 | 160 | 121 | 88 | 68 | 36 | 30 | 12 | 6 | 2 | 3 |
| Conduct problems | 123 | 226 | 162 | 96 | 47 | 25 | 7 | 3 | 3 | 0 | 0 |
| Hyperactivity/Inattention | 38 | 68 | 100 | 110 | 107 | 125 | 67 | 45 | 23 | 7 | 2 |
| Peer-relationship problems | 102 | 162 | 161 | 126 | 78 | 35 | 12 | 13 | 2 | 1 | 0 |
| Prosocial behavior | 2 | 1 | 3 | 3 | 17 | 74 | 91 | 101 | 142 | 153 | 105 |
|  | Parents | | | | | | | | | |  |
| Sum score | 0 | 1 | 2 | 3 | 4 | 5 | 6 | 7 | 8 | 9 | 10 |
| Emotional problems | 219 | 138 | 101 | 74 | 65 | 45 | 20 | 14 | 7 | 5 | 4 |
| Conduct problems | 149 | 167 | 147 | 96 | 71 | 33 | 20 | 6 | 2 | 1 | 0 |
| Hyperactivity/Inattention | 103 | 75 | 95 | 116 | 84 | 77 | 52 | 36 | 20 | 15 | 19 |
| Peer-relationship problems | 234 | 139 | 116 | 84 | 56 | 28 | 21 | 8 | 1 | 1 | 4 |
| Prosocial behavior | 2 | 0 | 1 | 9 | 15 | 44 | 81 | 121 | 128 | 142 | 149 |

**Psychosomatic complaints (N = 379)**

|  | Children | | | | | Parents | | | | |
| --- | --- | --- | --- | --- | --- | --- | --- | --- | --- | --- |
| Response category^a^ | 1 | 2 | 3 | 4 | 5 | 1 | 2 | 3 | 4 | 5 |
| Headache | 244 | 65 | 47 | 14 | 9 | 186 | 139 | 37 | 13 | 4 |
| Stomach ache | 270 | 66 | 20 | 14 | 9 | 206 | 126 | 31 | 12 | 4 |
| Back pain | 286 | 40 | 28 | 15 | 10 | 281 | 69 | 17 | 6 | 6 |
| Depressive mood | 276 | 63 | 24 | 11 | 5 | 237 | 110 | 18 | 13 | 1 |
| Nervousness | 269 | 57 | 37 | 11 | 5 | 234 | 96 | 35 | 12 | 2 |
| Irritability | 185 | 92 | 65 | 27 | 10 | 97 | 127 | 105 | 38 | 12 |
| Problems falling asleep | 211 | 63 | 48 | 27 | 30 | 189 | 119 | 45 | 17 | 9 |
| Dizziness | 323 | 34 | 15 | 6 | 1 | 348 | 24 | 6 | 1 | 0 |

^a^Response categories: 1 = never/rarely, 2 = once per month, 3 = nearly every week, 4 = several times per week, 5 = nearly every day.

**School grades (N = 458)**

|  | Children | | | | | | Parents | | | | | |
| --- | --- | --- | --- | --- | --- | --- | --- | --- | --- | --- | --- | --- |
| Response category^a^ | 1 | 2 | 3 | 4 | 5 | 6 | 1 | 2 | 3 | 4 | 5 | 6 |
| Mathematics | 66 | 193 | 156 | 39 | 4 | 0 | 67 | 195 | 151 | 43 | 2 | 0 |
| German | 76 | 250 | 114 | 18 | 0 | 0 | 75 | 240 | 128 | 15 | 0 | 0 |
| Physical education | 134 | 211 | 94 | 19 | 0 | 0 | 128 | 216 | 96 | 18 | 0 | 0 |
| Foreign language | 102 | 232 | 99 | 24 | 1 | 0 | 98 | 223 | 109 | 26 | 2 | 0 |

^a^Response categories: 1 = very good, 2 = good, 3 = satisfactory, 4 = sufficient, 5 = deficient,
6 = insufficient.
